# Hungarian validation of the Buss–Perry Aggression Questionnaire—Is the short form more adequate?

**DOI:** 10.1002/brb3.2043

**Published:** 2021-01-24

**Authors:** Szabolcs Zimonyi, Krisztian Kasos, Zsuzsa Halmai, Luca Csirmaz, Helga Stadler, Sándor Rózsa, Anna Szekely, Eszter Kotyuk

**Affiliations:** ^1^ MTA‐ELTE Lendület Adaptation Research Group Institute of Psychology ELTE Eötvös Loránd University Budapest Hungary; ^2^ Doctoral School of Psychology ELTE Eötvös Loránd University Budapest Hungary; ^3^ Bhaktivedanta College Budapest Hungary; ^4^ Institute of Psychology Károli Gáspár Református University Budapest Hungary

**Keywords:** aggression, Buss–Perry Aggression Questionnaire, factor analysis, Hungarian validation

## Abstract

**Objective:**

We aim to provide a publicly available Hungarian version of the BPAQ; compare the BPAQ factors to other personality traits; and compare both the original BPAQ factor structure provided by Buss and Perry (*J. Pers. Soc. Psychol*., *63*, 1992, 452), the revised BPAQ‐SF factor structure by Bryant and Smith (*J. Res. Pers*., *35*, 2001, 138), and the BAQ by Webster et al. (*Aggress. Behav., 40*, 2014, 120).

**Methods:**

The validation of the Hungarian version of the BPAQ was carried out on a Hungarian university sample (*N* = 841). There were three main focuses of data analysis: descriptive statistics, correlations, and confirmatory factor analyses.

**Results:**

CFA‐related statistics showed an adequate fit for the BPAQ 4 factors; however, contrary to prior validations of BPAQ, we were not able to clearly define the verbal aggression factor. We found that the shorter form of the BPAQ has a better model fit on our sample than the original form, while the model fit of the BAQ was in‐between these. BPAQ scales showed low to moderate relationship with the Barratt Impulsivity Scale and Hospital Anxiety and Depression Scale.

**Conclusion:**

Both the BPAQ and the BPAQ‐SF, also the BAQ provide acceptable model fitting on a Hungarian sample of university students. While most of BPAQ items provided adequate loadings on their hypothesized factors, two items (21 and 27) did not. We argue this is the result of conceptual inaccuracy of the original items.

## Graphical abstract

Both the BPAQ, the BPAQ‐SF and BAQ provide acceptable model fitting on a Hungarian sample of university students. While most of BPAQ items provided adequate loadings on their hypothesized factors, two items (21 and 27) did not. We argue this is the result of conceptual inaccuracy of the original items.

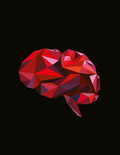

## 1 INTRODUCTION

Aggression is defined as purposeful behavior to inflict physical or psychological harm (American Psychological Association, 2007). Even though aggression is a basic human behavior and has been in the focus of psychological studies for decades, the conceptualization of this behavior still has its challenges. For example, Parrott and Giancola (2007) addressed “the criterion problem” of aggressive behavior: what exactly is aggression psychologically, and how we can categorize it. Their classification of aggression was based on a new taxonomic system. The five subtypes of Aggression were as follows: physical aggression, verbal aggression, postural aggression, damage to property and theft. These could be expressed actively or passively/directly or indirectly.

The Buss–Perry Aggression Questionnaire (BPAQ) is one of the gold standards in terms of measures of self‐report aggression. The BPAQ is derived from the Buss‐Durkee Hostility Inventory (BDHI). The BDHI (Buss & Durkee, 1957) was developed in the 1950s and remained in use for decades. The BDHI measures 7 dimensions of aggression (Assault, Indirect Hostility, Irritability, Negativism, Resentment, Suspicion, and Verbal Hostility). The BDHI was criticized for its lack of discriminant validity (Biaggio, 1981), its unstable factor loadings (Buss & Perry, 1992) and because it was found to strongly correlate with social desirability (Biaggio et al., 1981). Thus, a new questionnaire (BPAQ) was developed.

The BPAQ is a 29 item five‐point Likert scale inventory replacing the true or false type questions of the BDHI. It measures four factors of aggression: Verbal Aggression, Physical Aggression, Anger, and Hostility. The BPAQ has been in use in different languages and its psychometric properties appear to substantiate the original four‐factor model of aggression that was proposed by Buss and Perry. The 29 item BPAQ has gone through some changes since its inception. For example, Archer et al. (1995) criticized the questionnaire for the difficulty of obtaining classically “pure” measures, like the ones proposed by Buss and Perry. Some criticized the 29 item BPAQ because the four‐factor model explained only a portion of the common variance, model fit was only moderate (Bryant & Smith, 2001; Harris, 1995; Williams et al., 1996).

This criticism was led by Bryant and Smith (2001), who suggested to remove items from the questionnaire to improve its psychometric properties. The result is a 12 item BPAQ with better psychometric properties than the original 29 item scale. The Buss–Perry Aggression Questionnaire—Short Form (BPAQ‐SF) aimed to improve the common explained variance of the BPAQ (for which variance explained was only 80%). By omitting seventeen items from the original questionnaire, 12 items remained. This reduced form produced four conceptually similar factors as before: Anger, Physical Aggression, Hostility, and Verbal Aggression. Confirmatory Factor Analyses of the BPAQ‐SF supported the new model, fit was satisfactory on independent data sets (Diamond & Magaletta, 2006; Gerevich et al., 2007; Reyna et al., 2011; Webster et al., 2014).

Webster et al. (2014) also proposed a 12‐item model, the Brief Aggression Questionnaire (BAQ). This questionnaire gives an alternative for the BPAQ‐SF. The items were selected also from the BPAQ, using multiple criteria (item‐total correlations, factor loadings from principal axis factoring PAF, confirmatory factor analyses, wording and face validity of items). Contrary to the BPAQ‐SF, they kept the reversed items. While Bryant and Smith (2001) used 5 moderate samples to develop the BPAQ‐SF, Webster et al. (2014) had one larger sample for selecting the ideal items. The BAQ shows the same factor structure as the BPAQ and BPAQ‐SF. Their model outperformed the BPAQ‐SF in validity test but underperformed in internal consistency.

The BPAQ has been translated to various languages with adequate psychometric properties. There are translations available in Dutch (Meesters et al., 1996; Morren & Meesters, 2002), Japanese (Nakano, 2001), Spanish (Alvarado et al., 2007; García‐León et al., 2002; Santisteban & Alvarado, 2009), German (von Collani & Werner, 2005), Chinese (Maxwell & Maxwell, 2007), Pakistani (Iftikhar & Malik, 2014), and many others. A Hungarian version was also validated on a representative sample. Gerevich et al. (2007) conducted a study on a representative (*N* = 1,200) Hungarian sample. They concluded that the four‐factor model originally proposed by Buss and Perry is valid. The factor loadings, however, were not as clear as they were in the original BPAQ, and they found the BPAQ‐SF more adequate. However, this Hungarian translation is not publicly available.

Furthermore, aggression has been connected to various other personality traits, such as impulsivity, depression, and anxiety. Impulsiveness was found to be correlated with most BPAQ scales (Fossati et al., 2002; Güleç et al., 2008; Ramírez & Andreu, 2006; Vigil‐colet & Codorniu‐raga, 2015, 2004).

In sum, the BPAQ is a widely used tool to assess aggression, translated to many different languages. It has been previously translated to Hungarian, showing adequate psychometric properties (Gerevich et al., 2007), however, during our backward translation process certain translation errors were noticed. Thus, we made a modified Hungarian BPAQ, which we also provide here, as the first publicly available Hungarian version of the full Buss‐Perry Aggression Questionnaire (see BPAQ at Appendix 1) and the shorter versions as well (see BPAQ‐SF at Appendix 2, and BAQ at Appendix 3). We also aim to compare the factors of Aggression to other personality traits: Impulsivity, Anxiety, and Depression. Furthermore, there are some studies suggesting that shorter forms of the original 29 item BPAQ might have better psychometric properties. Thus, the purpose of this study was to attempt to remedy the issue of factor loadings: comparing both the original BPAQ factor structure provided by Buss and Perry (1992) and the revised BPAQ‐SF factor structure by Bryant and Smith (2001) and also investigating the BAQ by Webster et al. (2014) in our Hungarian university sample.

## 2 METHODS

### 2.1 Participants and procedure

The study protocol was approved by the Institutional Ethical Board. Recruitment took place in four different educational institutions (*N* = 841, Mean age = 23.55 *SD* = 8.04). Inclusion criteria were no present or past psychiatric illnesses. Participants received no compensation for participation. Participants were all Caucasian college and university students (53.5% female and 46.5% male).

The minimum target sample size for the confirmatory factor analysis was based on the “rule of thumb” which suggest 10 participants per item (10 × 29 = 290), and we duplicated this to be able to test the planned two models (BPAQ and BPAQ‐SF), resulting in a planned minimum sample size of 600 participants.

All participants provided written informed consent and filled out the Hungarian translation 29 item BPAQ questionnaire. Data collection the BPQA was performed in two waves. Instruction and statements of the questionnaire were exactly the same in both, however, in the first wave of data collection (80.1% of the total sample; *n* = 674) the answers were given on a seven‐point Likert scale. In the second wave of the collection, we used a five‐point Likert scale (19.8%; *n* = 167). In both cases, the answers ranged between “absolutely not true” to “absolutely true.” For the analysis, we transformed the seven‐point Likert scale data to five‐point Likert scale data. Reason behind reduction to five‐point scale was to simplify data analysis. When converting items from 7‐ to 5‐point scales, we used 0.66 step increments (1 = 1; 2 = 1.66; 3 = 2.33; 4 = 3; 5 = 3.66; 6 = 4.33; 7 = 5). t Tests were performed to compare BPAQ and BPAQ‐SF scales on the 5‐ and 7‐point Likert scales (see Tables 1 and 2). In most cases, the difference between the scale mean of the 5‐point Liker scale version and the 7‐point Likert scale version was not significant, and even in cases where the difference reached the level of significance, the differences in the means were rather low.

**Table 1:** Descriptive statistics of the 5‐ and 7‐point Liker scale measurements

BPAQ	*n* = 167	Mean	*SD*	BPAQ	*n* = 674	Mean	*SD*	BPAQ	Mean difference	Cohen's *D*
	BPAQ 5 total	2.22	0.5		BPAQ 7 total	2.3	0.57		0.08	0.15
	Anger 5	2.27	0.72		Anger 7	2.3	0.81		0.03	0.04
	PhysAgr 5	1.95	0.72		PhysAgr 7	2.03	0.74		0.08	0.12
	Hostil 5	2.07	0.63		Hostil 7	2.21	0.71		0.14	0.20
	VerbAgr 5	2.88	0.63		VerbAgr 7	2.93	0.67		0.05	0.08
BPAQ‐SF	*n* = 167	Mean	*SD*	BPAQ‐SF	*n* = 674	Mean	*SD*	BPAQ‐SF	Mean difference	Cohen's *D*
	BPAQ‐SF 5 total	2.03	0.56		BPAQ‐SF 7 total	2.11	0.64		0.08	0.13
	Anger 5	2.1	0.85		Anger 7	2.08	0.87		−0.02	0.02
	PhysAgr 5	1.6	0.82		PhysAgr 7	1.77	0.88		0.17	0.19
	Hostil 5	2.05	0.83		Hostil 7	2.21	0.91		0.16	0.19
	VerbAgr 5	2.39	0.72		VerbAgr 7	2.39	0.78		0	0.01
BAQ	*n* = 167	Mean	*SD*	BAQ	*n* = 674	Mean	*SD*	BAQ	Mean difference	Cohen's *D*
	BAQ 5 total	2.26	0.55		BAQ 7 total	2.36	0.6		0.10	0.18
	Anger 5	2.03	0.75		Anger 7	2.04	0.82		0.01	0.16
	PhysAgr 5	1.96	0.97		PhysAgr 7	2.14	1.02		0.18	0.17
	Hostil 5	2.06	0.79		Hostil 7	2.19	0.84		0.13	0.15
	VerbAgr 5	2.98	0.69		VerbAgr 7	3.08	0.71		0.10	0.14

**Table 2:** Comparison of 5‐ and 7‐point Likert scale ratings

	BPAQ					
		BPAQ 5 total	Anger 5	PhysAgr 5	Hostil 5	VerbAgr 5
BPAQ	BPAQ 7 total	n.s.				
	Anger 7		n.s.			
	PhysAgr 7			n.s.		
	Hostil 7				*p* = .02	
	VerbAgr 7					n.s.
	BPAQ‐SF					
		BPAQ_SF 5 total	Anger 5	PhysAgr 5	Hostil 5	VerbAgr 5
BPAQ‐SF	BPAQ‐SF 7 total	n.s.				
	Anger 7		n.s.			
	PhysAgr 7			*p* = .02		
	Hostil 7				*p* = .02	
	VerbAgr 7					n.s.
	BAQ					
		BAQ 5 total	Anger 5	PhysAgr 5	Hostil 5	VerbAgr 5
BAQ	BAQ 7 total	*p* = .03				
	Anger 7		n.s.			
	PhysAgr 7			*p* = .04		
	Hostil 7				n.s.	
	VerbAgr 7					n.s.

### 2.2 Questionnaires

The BPAQ explores aggression on four subscales: Anger (items 1, 9, 12, 18, 19, 23, 28), Physical Aggression (items 2, 5, 8, 11, 13, 16, 22, 25, 29), Hostility (items 3, 7, 10, 15, 17, 20, 24, 26), and Verbal Aggression (items 4, 6, 14, 21, 27). The 29 item BPAQ has two reversed items, one loading on the Anger (item 9) subscale and the other loading on Physical Aggression (item 16) subscale. Subscale scores were calculated by taking the mean of the appropriate items. The factors of the BPAQ‐SF remained conceptually the same (Appendix 2): Anger (items 12, 18, 28), Physical Aggression (items 11, 13, 25), Hostility (items 7, 17, 24), and Verbal Aggression (items 6, 21, 27). Subscale scores were calculated by taking the mean of the appropriate items. The psychometric properties of the first Hungarian version of the BPAQ have previously been investigated (Gerevich et al., 2007) providing adequate reliability indexes. As part of the present study, we made further corrections on this first Hungarian translation of the BPAQ since our backward translation prevailed small inaccuracies in about half the items, and significant translation errors in about two items. This modified version of the BPAQ was applied in the present study to investigate its psychometric properties.

A common measure of impulsivity is the Barratt Impulsivity Scale (BIS, Barratt, 1959). The BIS has been through numerous revisions, now we use the 11th version (Patton et al., 1995), translated to Hungarian by Kapitány et al. (2019). This version consists of 30 items scored on a four‐point Likert scale: (a) rarely/never, (b) occasionally, (c) often, and (d) almost always/always. This questionnaire measures impulsivity and has three factors: Cognitive impulsiveness (Ic), Motor impulsiveness (Im), and Nonplanning impulsiveness (Inp).

The Hospital Anxiety and Depression Scale (HADS) (Zigmond & Snaith, 1983) measures anxiety and depression on an intermixed 7–7 items long questionnaire, respectively. It was translated to Hungarian by Muszbek et al. (2006). The HADS (Zigmond & Snaith, 1983) is a self‐report tool containing 14 items, 7 items for an Anxiety (Ax), and 7 items for Depression (Dp) scale containing straightforward and reversed items as well. It is also scored on a four‐point Likert scale, and while answers differ in wording, their content ranges from “not at all” to “most of the time.” For both questionnaires, subscale scores were calculated by taking the mean of the appropriate items.

### 2.3 Data analysis in R

The questionnaire has been analyzed two ways: considering the BPAQ factor structure of 29 items by Buss and Perry (1992); and the BPAQ‐SF factor structure of 12 items by Bryant and Smith (2001). Data analysis was conducted in the R (R. Team, 2013) statistical language, in the RStudio (R. Team, 2015, 2004) software.

There were three main focuses of data analysis: descriptive statistics, correlations, and confirmatory factor analyses. For variable selection, the *sqldf* package (Grothendieck & Grothendieck, 2017), and for plotting the *corrplot* (Wei et al., 2017) and *ggplot2* (Wickham, 2011) packages were used. The *alpha* function of the *psych* (Revelle, 2012) package has been used to assess the internal consistency of the questionnaire scales. To test the normal distribution of the sample, the *shapiro.test* function of the *stats* package was used. Due to abnormal distribution of the variables, the Spearman correlation version of the *cor.tes*t function was used from the same *stats* package. To test the hypothesized factor structure of Buss and Perry (1992) on our sample, the *cfa* function of the *lavaan* package (Rosseel, 2012) was used for the confirmatory factor analysis. To see the distribution of our factor structure, a Maximum Likelihood factor analysis was conducted with the *factanal* function of the *stats* package. Prior to this, with the *eigen* function of the base package, we inspected the eigenvalues, and with the *nScree* and *plotnScree* functions of the *nFactors* (Raiche et al., 2010) package we visualized them. Gender differences of the dataframe were also inspected with the *t*.*test* function of *stats*. To test measurement invariance between 5‐ and 7‐point Likert Scale BPAQ groups and genders, we used the *measurementInvariance* function of *semTools*.

To test the hypothesized factor structure of Bryant and Smith (2001) as well, we used the same statistical methods as described above for the original factor structure. Instead of the four factors derived from the 29 items of the BPAQ, we used the newer four conceptually same factors of the BPAQ‐SF, derived from the 12 items Bryant and Smith recommended from the BPAQ. We also tested the 12‐item Brief Aggression Questionnaire (BAQ; Webster et al., 2014, 2015).

For both the original and the short versions of the BPAQ, we considered an item to be correctly loading on a factor if its factor loading was 0.3 on the hypothesized factor and also less than 0.3 on any other factor (see Buss & Perry, 1992).

## 3 RESULTS

### 3.1 Descriptive statistics

Mean age of the sample was 23.55 years (*SD* = 8.04). The gender distribution was slightly skewed toward women (53.86%). Mean score on the total Aggression questionnaire (BPAQ) was 2.29 (*SD* = 0.55), while the subscales had mean scores of 2.29 (*SD* = 0.79), 2.02 (*SD* = 0.74), 2.18 (*SD* = 0.70), and 2.92 (*SD* = 0.66) for the Anger (AN), Physical Aggression (PA), Hostility (HS), and Verbal Aggression (VA) subscales, respectively. The total mean score on the Barratt Impulsivity Scale (BIS) was 1.96 (*SD* = 0.33), while subscales had mean scores of 2.02 (*SD* = 0.37), 1.75 (*SD* = 0.37), and 2.05 (*SD* = 0.45) for the Nonplanning (Inp), Motor (Im), and Cognitive (Ic) subscales, respectively. Also, for the Hospital Anxiety and Depression Scale (HADS) the Anxiety (Ax) subscale mean score was 0.82 (*SD* = 0.52) and Depression (Dp) subscale mean score was 0.40 (*SD* = 0.40).

### 3.2 Internal consistency and correlations among aggression factors in the original and short form of the Aggression Questionnaire and Brief Aggression Questionnaire

Internal consistency was computed on for the subscales Anger, Physical Aggression, Hostility, and Verbal Aggression and the total mean score for the Aggression Questionnaire. Cronbach's alpha coefficient was used to determine reliability of the scales (Table 3). The alpha coefficients of the BPAQ were .90 (BPAQ), .84 (AN), .85 (PA), .80 (HS), and .64 (VA). The same values were .90 (BPAQ‐SF) .73 (AN), .74 (PA), .72 (HS), and .62 (VA) for the BPAQ‐SF and .79 (BPAQ‐SF) .73 (AN), .79 (PA), .65 (HS), and .51 (VA) for the BAQ.

**Table 3:** Internal consistency of the Buss–Perry Aggression Questionnaire (BPAQ)

Factor	Items of the BPAQ	Cronbach's alpha (*n* = 841)	
		Item‐total correlation if item deleted	Reliability for subscales
Anger (AN)	Some of my friends think I'm a hothead	.82	.84
	I am an even‐tempered person	.83	
	I flare up quickly but get over it quickly	.82	
	I have trouble controlling my temper	.79	
	When frustrated, I let my irritation show	.82	
	I sometimes feel like a powder keg ready to explode	.8	
	Sometimes I fly off the handle for no good reason	.82	
Physical aggression (PA)	If I have to resort to violence to protect my rights, I will	.83	.85
	I have become so mad that I have broken things	.84	
	Once in a while I can't control the urge to strike another person	.83	
	I have threatened people I know	.84	
	Given enough provocation, I may hit another person	.81	
	I can think of no good reason for ever hitting a person	.85	
	If somebody hits me, I hit back	.83	
	There are people who pushed me so far that we came to blows	.82	
	I get into fights a little more than the average person	.83	
Hostility (HS)	When people are especially nice, I wonder what they want	.78	.80
	I wonder why sometimes I feel so bitter about things	.77	
	I am suspicious of overly friendly strangers	.8	
	I am sometimes eaten up with jealousy	.79	
	At times I feel I have gotten a raw deal out of life	.78	
	I sometimes feel that people are laughing at me behind my back	.76	
	Other people always seem to get the breaks	.76	
	I know that "friends" talk about me behind my back	.78	
Verbal aggression (VA)	I tell my friends openly when I disagree with them	.65	.64
	I can't help getting into arguments when people disagree with me	.54	
	When people annoy me, I may tell them what I think of them	.56	
	I often find myself disagreeing with people	.61	
	My friends say that I'm somewhat argumentative	.59	

Shapiro–Wilk test was conducted to assess normality of scales, which yielded significant deviation from the normal distribution for all scales. Due to these results, nonparametric Spearman correlations were used to investigate interscale correlations of the scales and subscales. All scales had low or moderate intercorrelation, see Table 4. We also tested the intercorrelation of the BPAQ‐SF and BAQ subscales, which also yielded significant results similar to the BPAQ (Table 4).

**Table 4:** Correlations among BPAQ, BPAQ‐SF, BAQ and BIS and HADS subscales

		BPAQ					BPAQ‐SF					BAQ					BIS			HADS	
		BPAQ total	Anger	PhysAgr	Hostil	VerbAgr	BPAQ‐SF total	Anger	PhysAgr	Hostil	VerbAgr	BAQ‐SF total	Anger	PhysAgr	Hostil	VerbAgr	Nonplanning impulsivity	Motor impulsivity	Cognitive impulsivity	Anxiety	Depression
BPAQ	BPAQ total		.74**	.63**	.66**	.55**	.93**	.76**	.64**	.6**	.69**	.94**	.74**	.63**	.66**	.55**	.30**	.39**	.40**	.40**	.38**
	Anger			.41**	.48**	.57**	.82**	.91**	.4**	.45**	.62**	.74**	.88**	.35**	.45**	.44**	.33**	.43**	.42**	.43**	.32**
	PhysAgr				.27**	.36**	.57**	.4**	.83**	.2**	.3**	.72**	.37**	.9**	.29**	.36**	.17**	.19**	.15**	n.s.	.16**
	Hostil					.33**	.71**	.45**	.3**	.85**	.44**	.63**	.48**	.24**	.9**	.19**	.17*	.24**	.37**	.53**	.52**
	VerbAgr						.65**	.52**	.31**	.27**	.86**	.68**	.47**	.32**	.32**	.86**	.22**	.28**	.25**	.18**	.10**
BPAQ‐SF	BPAQ‐SF total							.75**	.53**	.64**	.47**	.87**	.75**	.53**	.64**	.47**	.31**	.41**	.41**	.45**	.40**
	Anger								.38**	.41**	.57**	.69**	.82**	.33**	.41**	.41**	.27**	.40**	.38**	.36**	.27**
	PhysAgr									.27**	.36**	.69**	.35**	.87**	.31**	.29**	.17*	.22**	.16**	.11**	.21**
	Hostil										.33**	.52**	.45**	.17**	.72**	.14**	.18**	.22**	.32**	.52**	.50**
	VerbAgr											.62**	.56**	.25**	.42**	.55**	.27**	.35**	.34**	.28**	.21**
BAQ	BAQ total												.72**	.71**	.66**	.63**	.27**	.36**	.36**	.32**	.34**
	Anger													.30**	.45**	.34**	.33**	.40**	.43**	.37**	.47**
	PhysAgr														.25**	.33**	.15**	.18**	.13**	n.s.	.13**
	Hostil															.17**	.17**	.25**	.37**	.47**	.45**
	VerbAgr																.12**	.16**	.10**	n.s.	n.s.

** Level of significance is < .01 (2‐tailed).

* Level of significance is < .05 (2‐tailed).

### 3.3 Correlation with other traits

We tested the relationship of aggression to other personality traits, namely the three impulsivity factors (Im, Ic, Inp) of BIS and the depression and anxiety factors (De, Ax) of HADS. Internal consistency was also computed for these factors. The alpha coefficients were 0.61 (Im), 0.68 (Ic), 0.61 (Inp), 0.78 (Ax), and 0.75 (De). Spearman correlations were significant for the total questionnaire mean scores of the BPAQ and BIS (*r* = .42, *p* < .01), BPAQ and HADS (*r* = .43, *p* < .01), and HADS and BIS (*r* =.31, *p* < .01). Correlations of BIS and HADS subscales to BPAQ subscales are presented in Table 4. Most of the correlations were significant. The correlation analysis of the BPAQ and BIS subscales showed moderate relationships between the BPAQ total score, the Anger subscale, and all three BIS subscales (Nonplanning impulsivity, Motor impulsivity, Cognitive Impulsivity), while the correlations between the BPAQ Physical Aggression, Hostility, and Verbal Aggression subscales and BIS subscales were rather small or negligible. As regards the relationship between the BPAQ and HADS subscales, the analysis showed moderate relationships between BPAQ total score, Anger, Hostility subscale and both the HADS Anxiety and Depression scalsube. The correlations between Physical Aggression, Verbal Aggression, and the HADS subscales were small or negligible. In the case of BPAQ‐SF and BAQ, we got similar results to when we used the subscales of the BPAQ.

As regards the cross‐correlations between the subscales of the BPAQ, BPPAQ‐SF, and BAQ scales, the same subscales of the three questionnaire versions showed high correlations in most cases. The lowest correlation was observable in case of the Verbal Aggression subscale of the BPAQ‐SF and the BAQ (*r* = .55). The within correlation analysis of each version showed in most cases moderate to high correlations between the subscales. Correlational analysis of the subscales of the three versions prevailed low to high correlations. For example, in most cases, high correlations were observed between all three total scores and the subscales of all versions. The Anger, Verbal Aggression subscales showed mostly moderate correlations with the subscales, the Physical Aggression, and Hostility subscales showed mostly low to moderate correlations with the subscales. All cross‐correlations can be seen in Table 4.

### 3.4 Effects of gender

*t* Tests were used to test possible gender effects. Results show that males had higher scores on the BPAQ as a whole, *t* = 3.42, *p* < .01, Cohen's *d* = 0.24 (mean scores ± standard deviations were 2.36 ± 0.59 for males and 2.22 ± 0.52 for females) and Physical Aggression *t* = 11.43, *p* < .01, Cohen's *d* = 0.81 (mean scores ± standard deviations were 2.31 ± 0.77 for males and 1.76 ± 0.60 for females); while women had higher scores on Anger, *t* = −3.22, *p* < .01, Cohen's *d* = 0.22 (mean scores ± standard deviations were 2.20 ± 0.80 for males and 2.37 ± 0.77 for females). The analyses on the subscales of the BPAQ‐SF and BAQ yielded similar results.

### 3.5 Confirmatory factor analysis based on Buss and Perry (1992)

Next, we carried out a confirmatory factor analysis (CFA) to assess the validity of the Buss (1992) model on the present sample using the lavaan version 0.6‐3 in R. With Maximum Likelihood estimation, χ^2^(371) = 1,245.78, *p *< .001, the model fit was moderate, the Comparative Fit Index (CFI) was .82 and the Tucker‐Lewis Index (TLI) was .8. Root Mean Square Error of Approximation (RMSEA) was .077. The Standardized Root Mean Square Residual (SRMR) was .080, just on the edge of the universally used cut‐off point for good fit.

Then, we conducted a Maximum Likelihood Factor analysis to assess the factor loadings of each item. In this CFA analysis, we used varimax rotation and based on the original factor structure the number of factors were fixed as 4. Loading on each factor can be seen in Table 5. For each item, factor loadings in bold represent the factor hypothesized based on the BPAQ.

**Table 5:** Confirmatory factor analysis based on Buss and Perry (1992)

Confirmatory factor	Items of the BPAQ	Factor loadings (*n* = 841)			
		Factor 1 (AN)	Factor 2 (PA)	Factor 3 (HS)	Factor 4 (VA)
Anger (AN)	I have trouble controlling my temper	**0.75**	0.25	0.21	0.03
	I sometimes feel like a powder keg ready to explode	**0.63**	0.27	0.29	0.03
	Sometimes I fly off the handle for no good reason	**0.60**	0.19	0.27	−0.06
	I flare up quickly but get over it quickly	**0.56**	0.16	0.11	0.18
	When frustrated, I let my irritation show	**0.56**	0.04	0.25	0.14
	Some of my friends think I'm a hothead	**0.54**	0.20	0.06	0.20
	I am an even‐tempered person^a^	**0.53**	0.09	0.22	−0.09
Physical aggression (PA)	Given enough provocation, I may hit another person	0.17	**0.83**	0.10	0.05
	There are people who pushed me so far that we came to blows	0.19	**0.75**	0.13	0.11
	If I have to resort to violence to protect my rights, I will	0.06	**0.61**	0.05	0.25
	At times I feel I have gotten a raw deal out of life	0.29	**0.61**	0.04	−0.09
	If somebody hits me, I hit back	0.06	**0.59**	0.09	0.32
	Once in a while I can't control the urge to strike another person	0.28	**0.55**	0.12	−0.15
	I can think of no good reason for ever hitting a person^a^	0.04	**0.47**	0.03	0.14
	I have threatened people I know	0.29	**0.42**	0.21	−0.08
	I have become so mad that I have broken things	0.46	**0.39**	0.21	−0.03
Hostility (HS)	Other people always seem to get the breaks	0.24	0.12	**0.67**	−0.10
	I sometimes feel that people are laughing at me behind my back	0.28	0.09	**0.66**	−0.11
	I wonder why sometimes I feel so bitter about things	0.27	0.00	**0.56**	−0.09
	At times I feel I have gotten a raw deal out of life	0.19	0.05	**0.55**	−0.04
	I know that "friends" talk about me behind my back	0.16	0.12	**0.54**	−0.05
	When people are especially nice, I wonder what they want	0.06	0.18	**0.52**	0.14
	I am sometimes eaten up with jealousy	0.27	−0.04	**0.46**	−0.07
	I am suspicious of overly friendly strangers	−0.03	0.11	**0.43**	0.19
Verbal aggression (VA)	I tell my friends openly when I disagree with them	0.12	0.08	−0.16	**0.52**
	When people annoy me, I may tell them what I think of them	0.28	0.23	0.10	**0.51**
	I can't help getting into arguments when people disagree with me	0.50	0.08	0.15	**0.28**
	I often find myself disagreeing with people	0.32	0.07	0.42	**0.23**
	My friends say that I'm somewhat argumentative	0.51	0.16	0.24	**0.16**

^a^ Marks revised items.

The main factor loadings are labeled with bold.

All Anger items load on the first factor (loadings range between 0.56 and 0.75). However, there are two verbal aggression items (0.51 and 0.50) and one Physical Aggression item (0.46) which also load on this factor. This Physical Aggression item also loads on the Physical Aggression factor (0.39). There are eight Physical Aggression items which clearly load on the Physical Aggression factor (loadings range between 0.42 and 0.83). Factor three is Hostility with mostly Hostility items (factor loadings range between 0.43 and 0.67), and one of the verbal aggression items (0.41). There are only two items that load on the Verbal Aggression factor above the 0.3 threshold (loadings are 0.51 and 0.52). In summary, there are three verbal aggression items (items 6, 21, 27) that seem to load on different factors.

### 3.6 Confirmatory factor analysis based on Bryant and Smith (2001)

The next analysis examined the model fit based on the shorter version of the Aggression Questionnaire (BPAQ‐SF), originally presented by Bryant and Smith (2001). This confirmatory factor analysis was conducted on the same dataframe as before, except this time it only contained mean scores of the twelve BPAQ‐SF items. Maximum Likelihood estimation was used, χ^2^(48) = 220.77, *p *< .001. The Comparative Fit Index improved greatly (CFI = .94), so did the Tucker‐Lewis Index as well (TLI = .92). Root Mean Square Error of Approximation (RMSEA) was .065, suggesting a good model fit. The Standardized Root Mean Square Residual (SRMR) was .052, lower than on the original scale, which also indicates a better fit. This model improved on the proportion of explained variance, which were 16% (AN), 14.1% (PA), 13.2% (HS), and 8.8% (VA). Factor loading on each factor can be seen on Table 6. Each item's hypothesized factor loadings are presented in bold. Factor loadings increased for most items compared to BPAQ factor structure, the loadings were as follows: 0.52–0.67 (AN), 0.39–0.92 (PA), 0.55–0.71 (HS), and 0.18–0.93 (VA). For the AN, PA, and HS factors each hypothesized items loaded correctly. However, for the Verbal Aggression factor only one item loaded correctly, contributing to most of the factors explained variance (0.93 factor loading). The other two items loaded on a different factor, AN – 0.30 and 0.56 on the AN, while only 0.25 and 0.18 on the VA factor.

**Table 6:** Confirmatory factor analysis based on Bryant and Smith (2001)

Confirmatory factor	Items of the BPAQ‐SF	Factor loadings (*n* = 841)			
		Factor 1 (AN)	Factor 2 (PA)	Factor 3 (HS)	Factor 4 (VA)
Anger (AN)	I have trouble controlling my temper	**0.67**	0.25	0.22	0.19
	Sometimes I fly off the handle for no good reason	**0.66**	0.15	0.24	0.07
	I flare up quickly but get over it quickly	**0.52**	0.18	0.12	0.15
Physical aggression (PA)	Given enough provocation, I may hit another person	0.17	**0.92**	0.03	0.04
	There are people who pushed me so far that we came to blows	0.19	**0.71**	0.11	0.09
	I have threatened people I know	0.29	**0.39**	0.20	0.04
Hostility (HS)	Other people always seem to get the breaks	0.19	0.14	**0.71**	0.11
	At times I feel I have gotten a raw deal out of life	0.14	0.08	**0.68**	0.02
	I wonder why sometimes I feel so bitter about things	0.22	0.04	**0.55**	0.09
Verbal aggression (VA)	I can't help getting into arguments when people disagree with me	0.33	0.09	0.10	**0.93**
	I often find myself disagreeing with people	0.30	0.11	0.29	**0.25**
	My friends say that I'm somewhat argumentative	0.56	0.14	0.21	**0.18**

The main factor loadings are labeled with bold.

### 3.7 Confirmatory factor analysis based on Webster et al. (2014)

We examined the 12‐item model proposed by Webster et al. (2014). Conducted on the same dataset as before, containing only the items proposed by the authors (Table 7). Maximum Likelihood estimation was used, χ^2^(48) = 346.16, *p *< .001. The Comparative Fit Index improved from the BPAQ but did not match the BPAQ‐SF (CFI = .89), the same could be observed with Tucker‐Lewis Index as well (TLI = .85). Root Mean Square Error of Approximation (RMSEA) was .086, worse than both BPAQ and BPAQ‐SF, suggesting a moderate model fit. The Standardized Root Mean Square Residual (SRMR) was .07, once again improved from the BPAQ, but did not match the BPAQ‐SF.

**Table 7:** Confirmatory factor analysis based on Webster et al. (2014)

Confirmatory factor	Items of the BAQ	Factor loadings (*n* = 841)			
		Factor 1 (AN)	Factor 2 (PA)	Factor 3 (HS)	Factor 4 (VA)
Anger (AN)	I am an even‐tempered person^a^	**0.53**	0.06	0.22	−0.02
	I have trouble controlling my temper	**0.72**	0.24	0.19	0.10
	Sometimes I fly off the handle for no good reason	**0.65**	0.15	0.23	0.02
Physical aggression (PA)	If I have to resort to violence to protect my rights, I will	0.09	**0.57**	0.06	0.20
	Given enough provocation, I may hit another person	0.18	**0.88**	0.11	0.01
	There are people who pushed me so far that we came to blows	0.18	**0.74**	0.18	0.07
Hostility (HS)	I sometimes feel that people are laughing at me behind my back	0.33	0.08	**0.58**	−0.09
	Other people always seem to get the breaks	0.24	0.03	**0.74**	−0.05
	When people are especially nice, I wonder what they want	0.16	−0.06	**0.94**	0.07
Verbal aggression (VA)	My friends say that I'm somewhat argumentative	0.55	0.13	0.24	**0.20**
	I tell my friends openly when I disagree with them	−0.01	−0.01	−0.11	**0.83**
	When people annoy me, I may tell them what I think of them	0.22	−0.11	0.09	**0.43**

^a^ Marks revised items.

The main factor loadings are labeled with bold.

### 3.8 Exploratory factor analysis of newly proposed models

As we have seen with the CFAs of the models proposed by Buss and Perry (1992; Table 5), Bryant and Smith (2001; Table 6), and Webster et al. (2014; Table 7), Verbal Aggression factors of each model had items with incorrect factor loadings. Namely, items 6, 21, and 27. Based on the metrics (item 6 contributed to most of the BPAQ‐SF’s VA factor, while also being conceptually more in line with the definition of VA), we investigated whether moving items 21 and 27 to the Anger factor (BPAQ and BPAQ‐SF), and moving item 27 to Anger (BAQ) resulted in improved model fit indices.

In the case of BPAQ, the exploratory model had similar model fit to the original model. Maximum Likelihood estimation was used, χ^2^(371) = 1,810.93, *p *< .001. The Comparative Fit Index remained similar (CFI = .83), the same could be observed with Tucker‐Lewis Index as well (TLI = .82). Root Mean Square Error of Approximation (RMSEA) was .068, slightly better then the original BPAQ. The Standardized Root Mean Square Residual (SRMR) was .07, once again slightly improved from the original BPAQ.

In the case of BPAQ‐SF, the exploratory model yielded improved results. Maximum Likelihood estimation was used, χ^2^(49) = 235.15, *p *< .001. The Comparative Fit Index improved greatly (CFI = .94), the same could be observed with Tucker‐Lewis Index as well (TLI = .92). Root Mean Square Error of Approximation (RMSEA) was .067, similar to the original BPAQ‐SF. The Standardized Root Mean Square Residual (SRMR) was .05, once again improved from the confirmatory BPAQ‐SF.

In the case of BAQ, the exploratory model yielded the most improved results. Maximum Likelihood estimation was used, χ^2^(48) = 196.25, *p *< .001. The Comparative Fit Index improved tremendously (CFI = .95), the same could be observed with Tucker‐Lewis Index as well (TLI = .93). Root Mean Square Error of Approximation (RMSEA) was .061, better than original BAQ. The Standardized Root Mean Square Residual (SRMR) was .05, once again greatly improved from the original BAQ.

### 3.9 Measurement invariance

When comparing groups, it is assumed that the measurement investigates the same underlying psychological construct in all groups (Milfont & Fischer, 2010; Webster et al., 2015). Using multiple group CFAs, we tested configural, metric (constraining factor loadings to be equivalent across groups), and scalar invariance (also constraining item intercepts alongside factor loadings to be equivalent across groups) to compare groups based on gender and whether they completed the 5‐ or 7‐point Likert scale BPAQ questionnaires. Configural invariance means the basic organization of the construct is supported in each group, while noninvariance means patterns of factor loadings differ between groups; metric invariance means fixing factor loadings across groups does not significantly alter model fit, while noninvariance means at least one model fit is different across groups, and its source should be investigated; scalar invariance means item intercepts are not significantly different across groups, while noninvariance means at least one item intercept is different across groups (Putnick & Bornstein, 2016). Configural invariance is measured by the overall model fit of the multiple group model, while we estimate both factor models simultaneously. If configural invariance is met, the metric invariance model is nested against the configural invariance model. If metric invariance is also met; then, the scalar invariance model is nested against the metric invariance model. Evaluation of measurement invariance is the subject of debate; however, the significance of change in χ^2^ or a −0.01 change in CFI for nested models is commonly used metrics.

As seen in Table 8, configural invariance is met in the case of BPAQ and BPAQ‐SF and BAQ models, gender, and item‐wise. In this section, the metric of significance of change in χ^2^ is used to determine measurement invariance. In case of gender, metric noninvariance can be observed in BPAQ and BAQ, and scalar noninvariance in BPAQ‐SF. Results suggest gender differences influence our model.

**Table 8:** Measurement invariance of BPAQ, BPAQ‐SF and BAQ

Models	χ^2^	*df*	CFI	TLI	RMSEA
BPAQ	1,245.78	371	.82	.8	.078
BPAQ: 5 vs. 7‐point scales					
1. Configural invariance	2,335.3	742	.82	.8	.071
2. Metric invariance	2,359.5^n.s^	767	.82	.81	.07
3. Scalar invariance	2,405.4*	792	.82	.81	.07
BPAQ: Gender					
1. Configural invariance	2020.7	742	.83	.81	.069
2. Metric invariance	2,293.3*	767	.82	.81	.069
3. Scalar invariance	2,488.96*	792	.8	.79	.071
BPAQ‐SF	220.77	48	.94	.92	.065
BPAQ‐SF: 5 vs. 7‐point scales					
1. Configural invariance	288.32	96	.94	.91	.069
2. Metric invariance	297.41^n.s^	104	.94	.92	.067
3. Scalar invariance	300.82^n.s^	112	.94	.94	.063
BPAQ‐SF: Gender					
1. Configural invariance	282.97	96	.94	.91	.068
2. Metric invariance	292.98^n.s^	104	.94	.92	.066
3. Scalar invariance	313.03*	112	.93	.92	.065
BAQ	346.16	48	.89	.85	.086
BAQ: 5 vs. 7‐point scales					
1. Configural invariance	398.45	96	.89	.85	.087
2. Metric invariance	410.05*	104	.89	.86	.084
3. Scalar invariance	423.16^n.s^	112	.89	.87	.081
BAQ: Gender					
1. Configural invariance	391.03	96	.89	.85	.085
2. Metric invariance	407.32*	104	.89	.86	.083
3. Scalar invariance	470.46*	112	.87	.85	.087

Abbreviation: n.s., not significant.

* *p* < .05.

Investigating the 5‐ and 7‐point scale models of BPAQ, metric invariance is seen, while scalar invariance is not met, indicating one intercept is not equal between the two groups. When comparing the 5‐ and 7‐point scale models of BPAQ‐SF and BAQ, scalar invariance is seen, indicating the difference between 5 and 7 item Likert scales on the BPAQ have no effect on our 12‐item models.

In this section, the metric of significance of a minimal −0.01 change in CFI for nested models is used to determine measurement invariance. Scalar noninvariance can be observed between gender groups in BPAQ and BPAQ‐SF and BAQ. In the case of comparing the two scale types, scalar invariance is obtained in all three, BPAQ, BPAQ‐SF, and BAQ. All models had similar measurement invariance across all measures.

## 4 DISCUSSION

This article investigated the model fitting of two factor structures based on the Buss–Perry Aggression Questionnaire (Buss & Perry, 1992). We also examined the relations of the BPAQ to other personality traits measured by questionnaires, namely Impulsivity (Barratt Impulsivity Scale; Barratt, 1959) and Anxiety and Depression (Hospital Anxiety and Depression Scale; Zigmond & Snaith, 1983). In line with previous findings (Fossati et al., 2002; Vigil‐colet & Codorniu‐raga, 2015, 2004), we found Nonplanning impulsivity, Motor Impulsivity, and Cognitive Impulsivity to be positively correlated with all BPAQ factors. We also found Depression to be correlated with all BPAQ factors, while Aggression correlated with every factor except the Physical Aggression factor. The strongest relationships were observed between Anger and Motor Impulsivity, Cognitive Impulsivity, Anxiety and between Hostility and Anxiety and Depression. Regarding gender differences, we found higher all‐around BPAQ mean scores and Physical Aggression mean scores higher for men, and higher Anger mean scores for women.

When testing the internal consistency of the three versions of the questionnaire, the total scale scores showed good and most of the subscales also showed adequate internal consistency values in all three versions. However, BAQ Verbal Aggression scale showed rather low Cronbach Alpha. The internal consistency of the subscales was generally lower in case of the shorter versions (BPAQ‐SF and BAQ) as compared to the BPAQ.

To test the model fit of the Buss–Perry Aggression Questionnaire (Buss & Perry, 1992) on our sample, we ran a confirmatory factor analysis (CFA). The chi‐square value and other CFA‐related statistics showed a bad fit; however, it is well known that the chi‐square statistics are sensitive to sample size. The results of the CFA were similar to the CFA results reported by others before (Collani & Werner, 2005; Gerevich et al., 2007; Santisteban & Alvarado, 2009).

Contrary to prior validations of the BPAQ, we were not able to clearly define the verbal aggression factor. Three items (items 6, 21, and 27) had higher factor loadings on a different factor (Anger) other than the target one (Verbal Aggression). Item 6 states “I can't help getting into arguments when people disagree with me,” item 21 states “I often find myself disagreeing with people,” while item 27 says “My friends say that I'm somewhat argumentative.” Item 6 seems adequate to the definition of verbal aggression. However, we argue that a person who fits the statements of item 21 and 27 could be viewed less as an aggressive, but rather an assertive individual. Like Gilbert and Allan (1994, p. 1) define, assertivity “refers to a number of different dimensions which include the ability to express self without anxiety, anger or aggression in various interpersonal situations, especially in situations of potential conflict of opinions, needs or rights.*”* Like Anderson and Martin (1995) stated, aggression and assertiveness can be so intertwined, that assertiveness can even be observed as a classificational factor of aggression. Disagreement and healthy argumentativeness, when viewed as a scale could mean either aggression or assertivity. Thus, the problematic items seem to be leaning toward assertivity. We propose modifications for items 21 and 27 to have a higher sense of aggression more in line with the modern perception of aggression and lean less toward assertivity. For example, Item 21: “I feel an urge to disagree with people.” Item 27: “My friends say that I always argue with people (even with no reason).”

After examining the BPAQ, we also tested the model fit of the factor structure hypothesized by Bryant and Smith (2001) on our sample. We found that the shorter form of the BPAQ has a better model fit on our sample than the original form. Based on the CFA‐related statistics, the Bryant and Smith version demonstrated a modest fit. The first three factors (AN, PA, and HS), each containing 3 items, had appropriate factor loadings and the hypothesized items loaded correctly. Our results are in line with previous literature (Reyna et al., 2011).

However, as in the case of the BPAQ, the items in the verbal aggression factor did not reach the cut‐off point. The VA factor of the BPAQ‐SF contained the three (on the BPAQ) problematic items, items 6 (“I can't help getting into arguments when people disagree with me.”), 21 (“I often find myself disagreeing with people.”), and 27 (“My friends say that I'm somewhat argumentative.”). We were interested in seeing how the shortened and modified structure of the questionnaire would affect the factor loadings and the distribution of these items. Item 6 this time had a very high factor loading, contributing to most of the factor's conceptual meaning. Item 21 and item 27 did not meet the required factor loading of 0.30 on the hypothesized factor, but had higher factor loading on Anger. These factor loadings paint a similar picture to the one we have seen on the BPAQ. On the original form, we found all of these items loading incorrectly. While we argued that items 21 and 27 did not fit the concept of verbal aggression well and were leaning more toward assertivity, we found item 6 to be in line with the definition of verbal aggression despite its incorrect factor loadings. In the case of the BPAQ‐SF factor structure, this argument seems to be consolidated. We can see that item 6 differs in terms of conceptual meaning from the other two, contributing to most of the VA factor of the BPAQ‐SF. These also underlie that items 21 and 27 should undergo some conceptual changes to fit better with the definition of verbal aggression.

Besides the BPAQ‐SF, we investigated the Brief Aggression Questionnaire proposed by Webster et al. (2014). BAQ proved to be the middle ground in terms of the model fit of the hypothesized factor structure. AN, PA, and HS factors had appropriate factor loadings once again, while the VA factor was the outlier. The Verbal Aggression factor is mostly defined by item 6 (“I can't help getting into arguments when people disagree with me.”), with a very high factor loading. The BAQ, however, did not contain the other two problematic items, 21 and 27. It could be hypothesized, that their absence would contribute to a more straightforward VA factor; however, this was not the case.

We also tested an exploratory model feasibility of the BPAQ, BPAQ‐SF, and BAQ with items 21 and 27 moved to the Anger factor. While BPAQ showed little to no improvement with the altered factor structures, BPAQ‐SF and mostly BAQ models improved drastically. In the case of BPAQ‐SF and BAQ, both CFI and TLI values changed for the better. It seems that the wording of these items 21 and 27 do indeed fit other scales more and are not in line with the previously introduced Verbal Aggression definitions. Once again, wording of these factors should be changed in future studies to be more in line with VA constructs. Besides these attempts, a wide range of further measurement practices (observational, self‐report, or laboratory) is available to gauge aggression (Suris et al., 2004) which could provide a great opportunity as a comparison to this questionnaire.

In conclusion, both the BPAQ, the BPAQ‐SF and the BAQ provided acceptable model fitting on a Hungarian sample of university students. However, the shorter form provides better model fitting in terms of measures of confirmatory factor analysis. The factors derived from the BPAQ‐SF explain a higher proportion of the common variance. While most of BPAQ items provided adequate loadings on their hypothesized factors, two items (21 and 27) did not. We argue this is the result of conceptual inaccuracy of the original items. Therefore, we suggest changing the wording, to better fit the concept of verbal aggression. Furthermore, a freely accessible version of the BPAQ (Appendix 1) is also provided to the Hungarian scientific community.

It also has to be noted that although the shorter versions showed clearer factor structures then the BPAQ, the internal consistency values were lower in case of the subscales of the shorter versions, suggesting that the reliability of the subscales did suffer from the item reduction. Thus, although the Cronbach alpha values are still around the acceptable range in case of the subscales of the short versions, interpreting scores of these subscales as independent scores should be handled with caution. They should rather be used as additional information on the pattern of aggression characteristics of the participants, like if example one has a high total score, scores on the subscales could give information on which facet it is mostly coming from.

## CONFLICT OF INTEREST

The authors (S. Zimonyi, K. Kasos, Z. Halmai, L. Csirmaz, H. Stadler, E. Kotyuk) declare that they do not have any interests which could constitute a real, potential or apparent conflict of interest with respect to his/her involvement in the publication. The authors also declare that they do not have any financial or other relations (e.g., directorship, consultancy, or speaker fee) with companies, trade associations, unions, or groups (including civic associations and public interest groups) that may gain or lose financially from the results or conclusions in the study.

## AUTHOR CONTRIBUTION

Eszter Kotyuk: Study concept and design. Zsuzsa Halmai and Eszter Kotyuk: Data collection. Szabolcs Zimonyi, Krisztián Kasos, Luca Csirmaz, Helga Stadler, Eszter Kótyuk, Anna Szekely, and Sandor Rozsa: Analysis and interpretation of the data. Szabolcs Zimonyi, Krisztián Kasos, Luca Csirmaz, Helga Stadler, Eszter Kótyuk, Anna Szekely, and Sándor Rózsa: Manuscript preparation. Eszter Kotyuk and Krisztian Kasos: Study supervision.

### PEER REVIEW

The peer review history for this article is available at https://publons.com/publon/10.1002/brb3.2043.

## Data Availability

The dataframe (df) and scripts of all the analyses are freely available at the Open Science Framework website (https://osf.io/afzb7/?view_only=e6ba90a3e59d450084788707e6ed27fe, Zimonyi et al., 2021). The dataframe has been stored as a comma separated Excel file and imported into R as such.

## Appendix 1 Hungarian version of the BPAQ Aggression Questionnaire ()

*Instrukció:* Kérjük, hogy az alábbi állításokkal kapcsolatban karikázza be azt a számot, amelyik a leginkább jellemzi Önt. 1 = egyáltalán nem jellemző; 2 = nem jellemző ; 3 = jellemző is meg nem is; 4 = jellemző; 5 = nagyon jellemző

**Table:**

A	1.	Néhány barátom úgy gondolja, forrófejű vagyok	1	2	3	4	5
PA	2.	Ha erőszakot kell alkalmaznom, hogy megvédjem a jogaimat, megteszem	1	2	3	4	5
H	3.	Amikor az emberek rendkívül kedvesek velem, azon tűnődöm, vajon mit akarnak	1	2	3	4	5
VA	4.	Nyíltan közlöm a barátaimmal, ha nem értek egyet velük	1	2	3	4	5
PA	5.	Előfordult már, hogy olyan dühössé váltam, hogy összetörtem dolgokat	1	2	3	4	5
VA	6.	Nem tudom visszafogni magam, hogy ne bonyolódjak vitába, amikor az emberek nem értenek velem egyet	1	2	3	4	5
H	7.	Csodálkozom azon, miért látom néha olyan sötéten a dolgokat	1	2	3	4	5
PA	8.	Néha képtelen vagyok visszafogni magam, hogy ne üssek meg egy másik embert	1	2	3	4	5
A^a^	9.	Kiegyensúlyozott, higgadt ember vagyok	1	2	3	4	5
H	10.	Bizalmatlan vagyok a túlságosan barátságos idegenekkel	1	2	3	4	5
PA	11.	Előfordult már, hogy megfenyegettem az ismerőseimet	1	2	3	4	5
A	12.	Gyorsan dühbe gurulok, de hamar le is higgadok	1	2	3	4	5
PA	13.	Ha kellően provokálnak, képes vagyok megütni másokat	1	2	3	4	5
VA	14.	Ha valaki bosszant, képes vagyok megmondani neki, hogy mit gondolok róla	1	2	3	4	5
H	15.	Néha nagyon mardos az irigység	1	2	3	4	5
PA^a^	16.	Nem hiszem, hogy lenne olyan ok, ami miatt megütnék egy másik embert	1	2	3	4	5
H	17.	Néha úgy érzem, keményen bánik velem az élet	1	2	3	4	5
A	18.	Nehezen tudom visszafogni az indulataimat	1	2	3	4	5
A	19.	Ha frusztrált vagyok, kimutatom az ingerültségemet	1	2	3	4	5
H	20.	Néha azt érzem, hogy az emberek kinevetnek a hátam mögött	1	2	3	4	5
VA	21.	Gyakran nem értek egyet másokkal	1	2	3	4	5
PA	22.	Ha valaki megüt, visszaütök	1	2	3	4	5
A	23.	Néha robbanásra kész puskaporos hordónak érzem magam	1	2	3	4	5
H	24.	Mintha mindig mások járnának jól	1	2	3	4	5
PA	25.	Előfordult, hogy egyes emberek addig provokáltak, amíg verekedésre nem került sor köztünk	1	2	3	4	5
H	26.	Tudom, hogy a „barátaim” kibeszélnek a hátam mögött	1	2	3	4	5
VA	27.	A barátaim szerint veszekedős vagyok	1	2	3	4	5
A	28.	Néha ok nélkül méregbe gurulok	1	2	3	4	5
PA	29.	Többször keveredek verekedésbe mint az átlagember	1	2	3	4	5

^a^ Marks revised items.

## Appendix 2 Hungarian version of the BPAQ Aggression Questionnaire Short Form (Buss & Perry, 1992)

*Instrukció:* Kérjük, hogy az alábbi állításokkal kapcsolatban karikázza be azt a számot, amelyik a leginkább jellemzi Önt. 1 = egyáltalán nem jellemző; 2 = nem jellemző ; 3 = jellemző is meg nem is; 4 = jellemző; 5 = nagyon jellemző

**Table:**

VA	6.	Nem tudom visszafogni magam, hogy ne bonyolódjak vitába, amikor az emberek nem értenek velem egyet	1	2	3	4	5
H	7.	Csodálkozom azon, miért látom néha olyan sötéten a dolgokat	1	2	3	4	5
PA	11.	Előfordult már, hogy megfenyegettem az ismerőseimet	1	2	3	4	5
A	12.	Gyorsan dühbe gurulok, de hamar le is higgadok	1	2	3	4	5
PA	13.	Ha kellően provokálnak, képes vagyok megütni másokat	1	2	3	4	5
H	17.	Néha úgy érzem, keményen bánik velem az élet	1	2	3	4	5
A	18.	Nehezen tudom visszafogni az indulataimat.	1	2	3	4	5
VA^a^	21.	Szükségét érzem annak, hogy ellentmondjak másoknak	1	2	3	4	5
H	24.	Mintha mindig mások járnának jól	1	2	3	4	5
PA	25.	Előfordult, hogy egyes emberek addig provokáltak, amíg verekedésre nem került sor köztünk	1	2	3	4	5
VA^a^	27.	A barátaim szerint folyton veszekszem másokkal (akár ok nélkül is)	1	2	3	4	5
A	28.	Néha ok nélkül méregbe gurulok	1	2	3	4	5

^a^ Marks revised items.

## Appendix 3 Hungarian version of the BAQ Aggression Questionnaire (Webster et al., 2014)

*Instrukció:* Kérjük, hogy az alábbi állításokkal kapcsolatban karikázza be azt a számot, amelyik a leginkább jellemzi Önt. 1 = egyáltalán nem jellemző; 2 = nem jellemző; 3 = jellemző is meg nem is; 4 = jellemző; 5 = nagyon jellemző

**Table:**

PA	2.	Ha erőszakot kell alkalmaznom, hogy megvédjem a jogaimat, megteszem	1	2	3	4	5
H	3.	Amikor az emberek rendkívül kedvesek velem, azon tűnődöm, vajon mit akarnak	1	2	3	4	5
VA	4.	Nyíltan közlöm a barátaimmal, ha nem értek egyet velük	1	2	3	4	5
A^a^	9.	Kiegyensúlyozott, higgadt ember vagyok	1	2	3	4	5
PA	13.	Ha kellően provokálnak, képes vagyok megütni másokat	1	2	3	4	5
VA	14.	Ha valaki bosszant, képes vagyok megmondani neki, hogy mit gondolok róla	1	2	3	4	5
A	18.	Nehezen tudom visszafogni az indulataimat	1	2	3	4	5
H	20.	Néha azt érzem, hogy az emberek kinevetnek a hátam mögött	1	2	3	4	5
H	24.	Mintha mindig mások járnának jól	1	2	3	4	5
PA	25.	Előfordult, hogy egyes emberek addig provokáltak, amíg verekedésre nem került sor köztünk	1	2	3	4	5
VA	27.	A barátaim szerint veszekedős vagyok	1	2	3	4	5
A	28.	Néha ok nélkül méregbe gurulok	1	2	3	4	5

^a^ Marks revised items.
